# PEG: Will It Come Back to You? Polyethelyne Glycol Immunogenicity, COVID Vaccines, and the Case for New PEG Derivatives and Alternatives

**DOI:** 10.3389/fbioe.2022.879988

**Published:** 2022-04-27

**Authors:** Yi Wen Kong, Erik C Dreaden

**Affiliations:** ^1^ David H. Koch Institute for Integrative Cancer Research, Massachusetts Institute of Technology, Cambridge, MA, United States; ^2^ Center for Precision Cancer Medicine, Massachusetts Institute of Technology, Cambridge, MA, United States; ^3^ Coulter Department of Biomedical Engineering, Georgia Institute of Technology and Emory University, Atlanta, GA, United States; ^4^ Department of Pediatrics, Emory School of Medicine, Atlanta, GA, United States; ^5^ Aflac Cancer and Blood Disorders Center, Children’s Healthcare of Atlanta, Atlanta, GA, United States; ^6^ Winship Cancer Institute of Emory University, Atlanta, GA, United States; ^7^ Petit Institute for Bioengineering and Bioscience, Georgia Institute of Technology, Atlanta, GA, United States

**Keywords:** polymer chemistry, drug delivery, nanotechnology, nanomaterials, PEG

## Introduction

**FIGURE 1:**
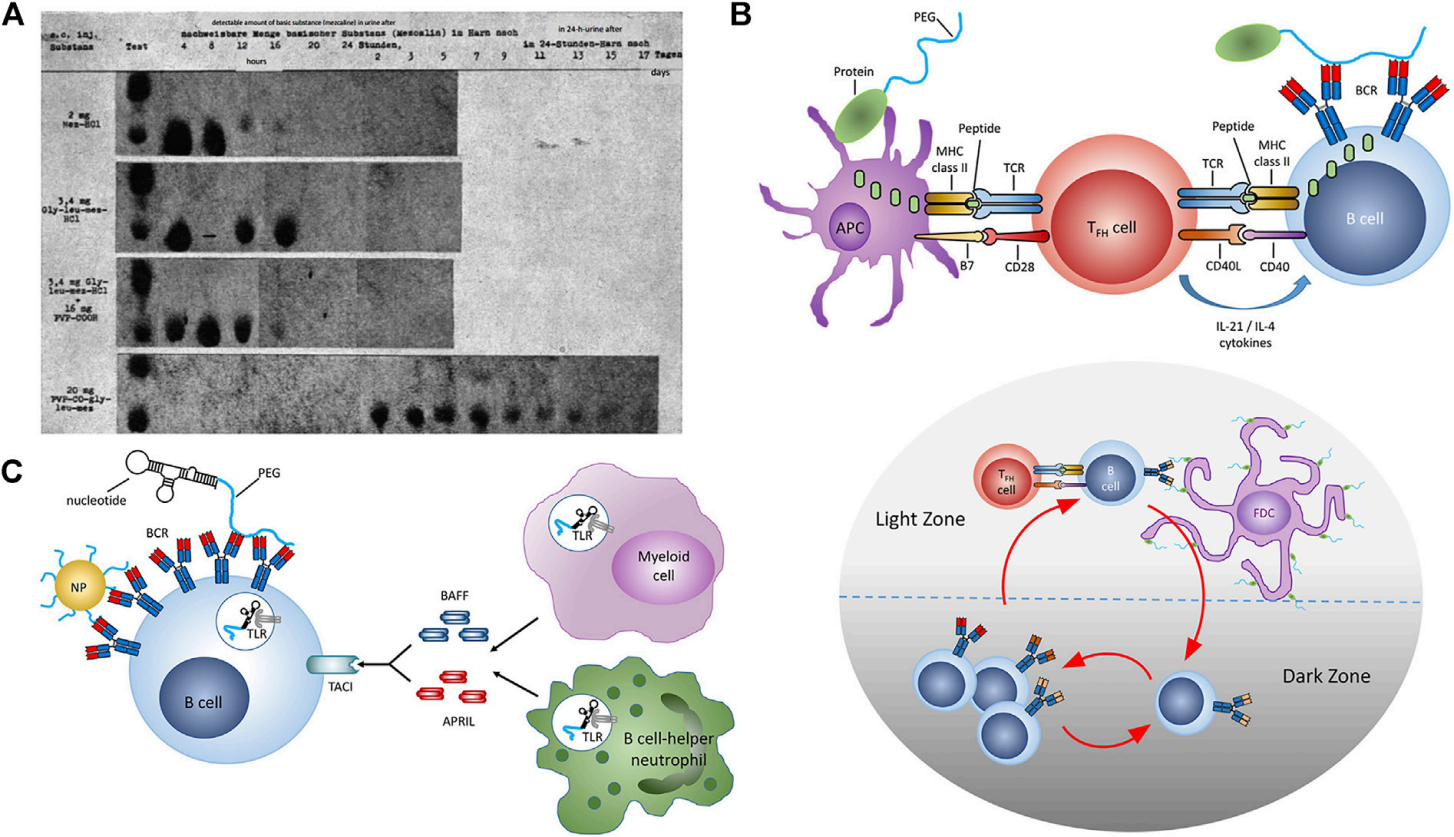
Polymer-Drug Conjugates: Inception to Immunology. **(A)** Renal excretion of mescaline and equimolar dosages of mescaline-PVP conjugate as measured by chromatography of urinary extracts obtained following s.c. adminisration in white mice circa 1955. **(B)** Thymus-dependent immune response against PEG. (upper panel) TFH activation following antigen presentation by APCs. Somatic hypermutation and class switching in B cells following antigen encounter and interaction with activated TFH cells. (lower panel) Affinity maturation of PEG-specific B cells in the spleen. **(C)** Thymus-independent immune response against PEG. Crosslinking of BCRs by PEG and coactivation of TLRs. Reproduced with permission from Reproduced with permission from **(A)** Luxenhofer, 2020 and **(B,C)** Chen et al., 2021. Copyright **(A)** 2020 de Gruyter GmbH and **(B,C)** 2021 American Chemical Society.

Polymer-drug conjugation (Harris, 1992; Harris and Chess, 2003; Haag and Kratz, 2006; Pelegri-O’Day et al., 2014; Hoffman, 2016; Ekladious et al., 2019) was first described in the 1954 by German chemist, Horst Jatzkewitz, who demonstrated that covalent attachment of poly (vinyl pyrrolidone) to the psychoactive compound, mescaline, could be used to prolong its circulation and duration of action (Figure 1A) (Jatzkewitz, 1954; Jatzkewitz, 1955; Luxenhofer, 2020). Yet despite its novelty and utility, Jatzkewitz’s innovation went largely unnoticed until the mid 1970s when it was revived by Ringsdorf, Kopecek, and Duncan, among others, who championed the notion that these novel macromolecules could enhance the suboptimal activity of various pharmaceuticals (Ringsdorf, 1975). It wouldn’t be until 1990—nearly 36 years from the publication of Jatzkewitz’s initial work—that the first polymer-drug conjugate would receive market approval in the form of Adagen, adenosine deaminase protein conjugated with 5 kDa poly (ethylene glycol), or PEG, used to treat a rare and hereditary, pediatric metabolic disorder called adenosine deaminase severe combined immunodeficiency (Hershfield et al., 1987).

Polymer-drug conjugates have since gradually increased in their clinical application, now with more than 29 marketed products that vary widely in polymer architecture (linear and branched), molecular weight (0.3–60 kDa per polymer), and degree of conjugation (1–69-82 per drug) and nearly all of which employ the synthetic polymer, PEG, a polyether typically produced by the ring-opening polymerization of ethylene glycol (Alconcel et al., 2011; Ekladious et al., 2019; Xu et al., 2022). In addition to the diversity of their appended polymers, these therapeutics also vary widely in their drug partner, ranging from: 1) peptides (e.g. Somavert HGH receptor antagonist) to 2) small molecules (e.g. SMANCS neocarzinostatin chemotherapy and Movanik naloxone laxative) and 3) nucleic acids (e.g. Macugen anti-VEGF aptamer antiangiogenic) (Perdue et al., 2020). More recently, polymer conjugation has demonstrated further clinical utility in stabilizing lipid nanoparticles used to deliver small interfering RNA (siRNA, Onpattro) for the treatment of hereditary transthyretin-mediated (hATTR) amyloidosis (Zhang et al., 2020), as well as both current mRNA-based vaccines for SARS-CoV-2 (COVID-19), BNT162b2/Comirnaty and mRNA-1273/Spikevax (Schoenmaker et al., 2021). Interestingly, all three nanoparticle formulations share in their use of lipids tethered with 2 kDa linear, methoxy-terminal PEG (mPEG). While Phase III clinical trials for both mRNA vaccines demonstrated overwhelming safety and efficacy (e.g. 4.7 and 2.8 anaphylactic reactions cases per million registered during the first months both vaccination campaigns, respectively (CDC COVID-19 Response Team and Food and Drug Administration, 2021)), their widespread use has led to concerns from some that pre-existing anti-PEG antibodies may induce hypersensitivity reactions (de Vrieze, 2021) or that drug-induced PEG immunity may impact the efficacy or safety of subsequently administered PEGylated drugs or vaccines.

PEG’s remarkable hydrophilicity, flexibility, inertness, and relative biocompatibility have found the polymer numerous uses beyond modulating drug circulation or activity and today it can be found near ubiquitously in both consumer products such as detergents, cosmetics, and car wax, as well as in industrial applications including electroplating, historical artifact preservation, and molded product production (Harris, 1992; Prime and Whitesides, 1993; Harris and Chess, 2003; Li et al., 2005; Jokerst et al., 2011). PEGylating has also been used to improve stability of contrast agents for *in vivo* fluorescence imaging, photodynamic therapy, and sonodynamic therapy (Ding et al., 2018; Chen et al., 2021; Xu et al., 2022). Given PEG’s near exclusive utilization in polymer-drug conjugates, our rapidly increasing consumer use of the compound, and recent, prevalent, and systemic exposure to PEG in the form of mRNA vaccines and boosters for SARS-CoV-2 (currently >0.5 bn doses (C ovid-data-tracker, 2022) in the United States) (Polack et al., 2020; Baden et al., 2021), several obvious questions arise with relevance to both public awareness and public health: *Is PEG immunogenetic? Does prior environmental exposure or PEG-drug conjugate therapy impact immune responses to PEG? Will PEG immunogenicity affect future vaccine efficacy? How can we minimize and hedge-against PEG immunogenicity in future polymer-drug formulations?*

### Immunity Towards PEG Is Pre-existing and Drug Exposure-inducible

PEG was classified as a GRAS (Generally Recognized as Safe) food ingredient by the FDA in 1973 and has a long history of safe use in humans. It is the most widely used stealth polymer in drug delivery and is typically regarded as a non-immunogenic polymer. Early studies by Richter and Akerblom in 1984 found that 0.2% of treatment-naïve individuals (individuals who have never received PEGylated biopharmaceuticals), had antibodies specific to PEG in their plasma (Richter and Åkerblom, 1984). Since then, the presence of pre-existing anti-PEG antibodies has been reported to range from 4.5 to 43.1% in treatment-naïve donors (Shpetner and Vallee, 1991; Garratty, 2004; Chen et al., 2016; Lubich et al., 2016; Yang et al., 2016), leading to the hypothesis that the frequency of pre-existing anti-PEG antibodies is increasing over time (Yang et al., 2016). Recent analysis of 79 historical (samples collected from the 1970s–1990s) and 377 contemporary human serum samples, indicate the presence of anti-PEG antibodies (IgG and IgM) in approximately 56 and 72% of samples respectively (Yang et al., 2016) with no significant difference in the measured concentrations of anti-PEG IgG and IgM, strongly suggesting that an apparent increase in pre-existing anti-PEG antibodies with time may in fact be a consequence of increased sensitivity in anti-PEG immunoassays developed in recent years (Yang et al., 2016; Chen et al., 2021). For example, direct binding assays using beads or ELISA plates are generally more sensitive compared to traditional bridging assays. Although these studies found that the prevalence of pre-existing anti-PEG antibodies was higher than was previously appreciated, the absolute concentrations of anti-PEG remain low in most positive individuals (Chen et al., 2021) and, as discussed later, drugs administered at different levels may be differentially impacted by pre-existing PEG immunity.

In addition to treatment-naïve immunity, anti-PEG antibodies are also drug-inducible and associated with systemic administration of PEGylated proteins (Chen et al., 2021), nucleic acids, liposomes, and nanoparticles (Judge and MacLachlan, 2008; Mima et al., 2015; Avci-Adali et al., 2013; Ishida et al., 2006a; Ishida et al., 2006b; Ishida et al., 2006c; Kozma et al., 2019). Drug-induced anti-PEG antibody responses occur via two principal mechanisms: T cell-dependent (TD) and T cell-independent (TI) pathways (Figures 1B,C). TD is typically associated with PEGylated proteins and peptides (Mima et al., 2015; Elsadek et al., 2020), while TI has been associated with systemic exposure to PEGylated nanoparticles (Freire Haddad et al., 2022). Anti-PEG antibodies induced by TD occur when peptides are presented by B cells to helper T cells, and is characterized by an initial peak of IgM, followed by class switching, and a larger peak in IgG (Freire Haddad et al., 2022). TI occurs when the antigen crosslinks receptors on IgM memory B cells and is characterized by high concentrations of IgM and low concentrations of IgG. Antibodies produced via the TI pathway have a weaker affinity for PEG compared to TD (Freire Haddad et al., 2022). While the basic underpinnings of anti-PEG immunity such as these are clear, 1) our understanding of how these processes vary with health or disease status, age, sex, or ethnicity and 2) our ability to predict the magnitude and functional impact of these responses on patients collectively remain unclear.

### PEG Immunity can Induce Hypersensitivity Reactions and Alter Drug Transport/Efficacy but these Effects Vary Across Formulation Type and Mode of Administration

Hypersensitivity reactions, including anaphylaxis has been reported in association with many PEG-containing formulations including PEG-protein conjugates (pegloticase (Lipsky et al., 2014), pegvaliase (Gupta et al., 2018), pegaspargase (Hasan et al., 2017; Browne et al., 2018; Liu et al., 2019), pegcrisantaspase (Rau et al., 2018)), PEG excipients (polysorbate 80 (Pérez-Pérez et al., 2011)), contrast agents (SonoVue (de Groot et al., 2004; Geleijnse et al., 2009)), liposomes encapsulating oligonucleotides or plasmid DNA (Semple et al., 2005; Judge et al., 2006), and liposomal doxorubicin (Chanan-Khan et al., 2003; Szebeni, 2014). Pre-existing PEG antibodies, in contrast, have been implicated in hypersensitivity reactions to PEGylated medicines including pegaspargase (Liu et al., 2019) and the RNA aptamer, pegnivacogin (Povsic et al., 2013). Acute severe allergic reactions to pegnivacogin were observed only in patients with pre-existing anti-PEG antibodies, and the level of anti-PEG IgG antibodies correlated with adverse event severity (Povsic et al., 2016). In addition, 2 of 25 phenylketonuria patients treated with pegvaliase developed anaphylactic and hypersensitivity reactions to a PEGylated contraceptive (Longo et al., 2014) and 3 patients who developed allergies to pegaspargase also experienced hypersensitivity reactions when treated with pegcrisantaspase (Rau et al., 2018), indicating that anti-PEG antibodies induced by one PEGylated medicines can cross-react to other subsequently administered PEGylated medicines. The mechanism(s) by which anti-PEG antibodies induce hypersensitivity reactions is poorly understood; however, some possible mechanisms by which pegylated nanoparticles and pegylated nucleotides could induce hypersensitivity reactions include: 1) complement activation-related pseudoallergy (CARPA) (Szebeni et al., 2011; Dézsi et al., 2014; Mohamed et al., 2019), whereby anti-PEG antibodies bound to PEG on a nanoparticle or liposome surface can activate the complement cascade, liberating the anaphylatoxins C3a and C5a (Neun et al., 2018; Mohamed et al., 2019; Chen et al., 2020) and 2) Fc receptor activation of innate immune cells either by anti-PEG IgE antibodies (Shah et al., 2013; Stone et al., 2019; Zhou et al., 2021) or allergen-specific IgG that binds to Fc gamma receptors (FcγRs) expressed on platelets, macrophages, basophils, or neutrophils to release various mediators such as platelet-activating factor (PAF), cysteinyl leukotrienes (CysLTs), histamine, and serotonin (Finkelman, 2007; Reber et al., 2017; Beutier et al., 2018).

Accelerated blood clearance (ABC) of PEGylated compounds was identified in mice in 1999, and in patients treated with pegaspargase in 2007 (Cheng et al., 1999; Cheng et al., 2000; Armstrong et al., 2007) and is caused by an immune reaction associated with repeat exposure to PEG. The first injection of PEGylated drugs induces anti-PEG antibodies, which then bind and form an immune complex with the second dose of the PEGylated compound to activate the complement system. This results in the opsonization of PEG with C3 fragments and enhanced uptake by Kupffer cells in the liver and can result in altered drug pharmacokinetics and biodistribution (PK, BD) and reduced drug efficacy in subsequent doses (Dams et al., 2000; Ishida et al., 2006a; Ishida et al., 2008; Ishida and Kiwada, 2008; Hashimoto et al., 2014). Rapid drug clearance and loss of drug efficacy have been reported following treatment with PEG-uricase, pegvaliase (Gupta et al., 2018), PEGylated liposomes (Dams et al., 2000; Laverman et al., 2001; Ishida et al., 2003), and PEGylated liposomal doxorubicin. ABC has also been observed in animal models treated with empty PEGylated liposomes (Dams et al., 2000; Semple et al., 2005; Ishida et al., 2006a; Ishida et al., 2006b), poly(lactic acid) (PLA) nanoparticles, microbubbles, and lipoplexes (Ishihara et al., 2009; Fix et al., 2018). In addition, anti-PEG antibodies can hinder the distribution of PEGylated nanoparticles to target tissues. For example, N-linked glycans present on anti-PEG antibodies bound to PEGylated nanoparticles can interact with mucin in the mucosal layer and prevent passage to epithelial surfaces (Henry et al., 2016).

Some PEGylated nanomaterials and proteins do not display ABC in animal models (Koide et al., 2008; Kaminskas et al., 2011; Koide et al., 2012; Grenier et al., 2018) and one explanation for this phenomenon is that in order for ABC to occur, a threshold molar ratio of anti-PEG antibodies to PEG compound is required for efficient clearance (Shiraishi et al., 2016; McSweeney et al., 2018). For example, the molar concentration of PEG-proteins in circulation is typically lower than that of PEG-liposomes (Grenier et al., 2018) at therapeutic dosing levels; thus, nanoparticles are thought to be less vulnerable to anti-PEG antibody-associated clearance than proteins. Indeed, prior studies show that strong ABC is observed when the number of antibodies in circulation exceeds the number of PEGylated compounds (Xu et al., 2022). This trend holds across most PEGylated compounds including proteins, liposomes, micelles, and polymeric nanoparticles and agrees with previous studies showing that three anti-PEG antibodies per PEGylated protein or about 10 anti-PEG antibodies per pegylated liposome are required for ABC (Shiraishi et al., 2016; McSweeney et al., 2018; Chang et al., 2019). These findings suggest that only compounds dosed at very low molar concentrations (*e.g.* PEG-IFNα) may be susceptible to polymer-specific ABC whereas the estimated threshold concentration of anti-PEG antibodies needed to accelerate the clearance of nucleic acid drug carriers (*e.g.* Patrisan) overwhelmingly exceed those observed in patient blood (Xu et al., 2022).

In addition to formulation-dependent susceptibility to polymer immunogenicity, mode of administration can also modulate the impact of antibody recognition. Most clinically approved polymer-drug conjugates are intravenously administered and thus their interaction with plasma IgG and IgM is higher than may be expected following intramuscular or intratumoral injection, as is common among many mRNA indications including both BNT162b2/Comirnaty and mRNA-1273/Spikevax (Schoenmaker et al., 2021). Thus, the strikingly low rates of anaphylaxis observed following SARS-CoV-2 mRNA vaccination (CDC COVID-19 Response Team and Food and Drug Administration, 2021) may be attributable in part to its intramuscular administration. Future studies focusing on the impact of polymer type/architecture/density and corresponding immunogenicity on drug efficacy and transport (*e.g.* lymphatic) following local administration are therefore warranted.

### PEG Immunogenicity can be Minimized but Alternative Polymers in Clinical Use are Lacking

Having established that PEG immunogenicity can limit the clinical utility of PEG-drug conjugates and that nanoparticle-based formulations may be less vulnerable to some of these effects relative to polymer-protein drug conjugates, how can one minimize the impact and risk of immunogenicity-diminished efficacy from future polymer-conjugated drugs and vaccines? As discussed above, PEG immunogenicity can arise through a variety of mechanisms (Xu et al., 2022) and includes antibody recognition associated with hypersensitivity reactions (e.g. anaphylaxis), accelerated blood clearance, premature drug release, or cross-reaction to other PEGylated therapies, among others. While limited in number, prior studies suggest that PEG antibody recognition is strongly dependent on polymer molecular weight (Xu et al., 2022), architecture, and end-functional group (Saifer et al., 2014). For example, antibodies with affinity towards backbone ethylene oxide units recognize immobilized PEG that is 2 kDa and larger with a minimum epitope subunit of approx. 16 repeats (700 Da) (Lee et al., 2020). Given that nearly all systemically administered polymer-drug conjugates are 2 kDa and above—per linear chain—the utilization of higher densities of lower molecular weight PEG may diminish the therapeutic impact of these backbone-specific antibodies. Such an approach is conceptually illustrated by branched PEG-drug conjugates (e.g. peginterferon alfa-2a, certolizumabpegol, and pegaptanib); however, those in clinical use (and which are systemically administrable) are limited to single site-modified, di-branched PEGs with per-arm molecular weight of approx. 10–30 kDa and with methoxy terminal groups; thus, the use of increasingly branched PEGs (i.e. hyperbranched, star, dendritic, bottlebrush) of lower per-branch molecular weight may diminish recognition by backbone-specific antibodies while maintaining favorable drug circulation, solubility, stability, activity profiles.

Polymer end-terminal groups can also play an important role in engineering future, less immunologically vulnerable PEG-drug conjugates as antibodies that recognize end-groups represent the other primary class of PEG-specific antibodies detected *in vivo*. While all clinical PEG-drug conjugates are chain-terminated by methoxy groups, recent preclinical studies suggest that hydroxy-terminal PEG conjugates generate lower amounts of backbone-specific anti-PEG IgM (Shimizu et al., 2018) and, while this improved immunogenicity comes with the tradeoff of higher complement activation and second-dose ABC (and typically, slightly shorter circulation half-life (Arvizo et al., 2011)), these findings may lead to the development of future polymer-drug conjugates with less propensity for immune activation. Other polymer end-group engineering strategies include the utilization of zwitterionic (Arvizo et al., 2011), ethoxy, and n-butyl ether (Saifer et al., 2014) moieties.

In addition to direct modifications of the polymer, corresponding drugs themselves can also modulate PEG immunogenicity. The introduction of 2’-fluro-modified pyrimidines and 2′-O-methyl-modifed purines has been shown to reduce the immunogenicity of PEGylated nucleic acids (Judge et al., 2005; Wang et al., 2009; Yu et al., 2009; Lee et al., 2016) while chemotherapeutics cytotoxic to B cells such as doxorubicin, mitoxantrone or oxaliplatin (Laverman et al., 2001; Ishida et al., 2006c; Cui et al., 2008; Abu Lila et al., 2012; Nagao et al., 2013) have been shown to mitigate anti-PEG IgM induced via PEGylated liposomal drug carriers often used to deliver these compounds *in vivo* (Cui et al., 2008; Mohamed et al., 2019).

Pharmacologic approaches have been further employed to diminish the impact of polymer immunogenicity including conjugation to or pre-treatment with immunosupressants, as well as the pre-treatment or co-infusion of tolerogenic compounds. Khanna et al. for example recently reported that pretreatment with the B/T cell immunosuppressant, mycophenolate mofetil, significantly improved treatment outcomes in a Phase I trial of patients with gout receiving pegloticase (Khanna et al., 2021). Other immunosuppressives under investigation to mitigate pegloticase immunogenicity include methotrexate, azathioprine, and leflunomide, while those used in conjunction with other ADA-prone therapies include rapamycin and anti-CD20. Likewise, pre-treatment or co-treatment with polymer, in particular high molecular weight (*i.e.* 40 kDa) PEG, has also been shown to reduce liposome-induced anti-PEG antibodies in preclinical studies (McSweeney et al., 2021). Taken together, these pharmacologic approaches are viewed by some to obviate the need PEG alternatives or derivatives; however, the deployment of immunosuppressives in combination with polymer-based vaccines and immunostimulatory therapies presents significant tradeoffs to drug efficacy, while PEG-based tolerogenics remain to be tested in patients.

Given 1) the therapeutic impact of PEG on drug immunogenicity, 2) the possible increasing prevalence of pre-existing and drug-induced PEG immunity, 3) the growing public need for safe and effective mRNA vaccines, and 4) our prevailing reliance on PEG for use in clinically approved nucleic acid and polymer-drug conjugate therapies (Schoenmaker et al., 2021), it is clear that the development and clinical validation of alternatives to (or derivatives of) PEG represents not only an unmet clinical need but also one with broad public health and national strategic interest. Indeed, the need for alternatives to PEG is a common refrain among those in the field (Harris, 1992), one as old as the first polymer-drug conjugate, Adagen; however, given the wide variety of potential candidate macromolecules such as polysaccharides, polyglycerols, and glycopolymers, (reviewed in detail elsewhere (Knop et al., 2010; Pelegri-O’Day et al., 2014; Bludau et al., 2017; Ekladious et al., 2019; Xu et al., 2022)), it begs the question as to why alternatives have yet to be approved (and studied post-approval) beyond poly(styrene co-maleic acid) (1993, Japan). Concerns over PEG immunogenicity have led some pharmaceutical companies to shy-away from or drop PEGylated products from their pipelines entirely (de Vrieze, 2020), thus the prospect of biopharma advancing clinically untested polymers through lengthy and expensive clinical trials is a difficult ask in the absence of a thoughtful incentive structure.

Given the challenging risk-reward of advancing non-PEG-based polymer-drug conjugates towards clinical translation, what can governments and funding agencies do to facilitate continued innovation in polymer-drug conjugate development and ensure the capacity for safe and effective vaccination at-scale? 1) Biosimilar-like regulatory guidelines for conjugable polymers (i.e. polysimilars) may be one approach to formalize and streamline the approval of new polymer-drug conjugates, albeit one likely requiring increased rigor given the wide structural diversity and potential health hazards of various polymer subunits relative to proteins. 2) Funding or federal lab support to perform large-scale longitudinal studies of immunogenicity towards polymers and other drug conjugates/excipients (lipids, polysaccharides, polypeptides, etc) would elucidate current (and potentially dynamic or age-, race-, and sex-specific) risks of polymer immunogenicity to human health, drug conjugate efficacy, and the strategic national need for mRNA vaccine-stabilizing polymers. 3) Federally subsidized R&D to offset the risks taken-on by companies exploring PEG- and other polymer-conjugates would greatly incentivize further innovation in this space. 4) Funding to improve our poor mechanistic understanding of polymer-induced immunogenicity and associated short- and long-term health risks would accelerate the discovery of new PEG derivatives and alternatives or propel historically utilized polymers through clinical translation. 5) Federal partnerships to ensure the financial viability of domestically manufactured, pharmaceutical-grade PEG and other polymers, as a matter of national interest, would ensure our readiness for future pandemics (and supply chain challenges) surely yet-to-come. In closing, while it is tempting to suggest a singular direction for polymer-drug conjugate development in the future, we also acknowledge that the ideal properties for a conjugation partner vary substantially with drug class, mode of administration, dosing frequency, and disease indication as discussed above; thus, with proper incentives, funding, and tools we anticipate that future conjugates will not only increase in diversity but also diverge based upon drug type and/or indication.
